# Impact of KvLQT1 potassium channel modulation on alveolar fluid homeostasis in an animal model of thiourea-induced lung edema

**DOI:** 10.3389/fphys.2022.1069466

**Published:** 2023-01-09

**Authors:** Mélissa Aubin Vega, Alban Girault, Damien Adam, Jasmine Chebli, Anik Privé, Émilie Maillé, Annette Robichaud, Emmanuelle Brochiero

**Affiliations:** ^1^ Centre de Recherche du Centre Hospitalier de l’Université de Montréal (CRCHUM), Montréal, QC, Canada; ^2^ Département de Médecine, Université de Montréal, Montréal, QC, Canada; ^3^ Laboratoire de Physiologie Cellulaire et Moléculaire (LPCM), Amiens, France; ^4^ SCIREQ Scientific Respiratory Equipment Inc., Montréal, QC, Canada

**Keywords:** potassium channels, pulmonary edema, animal model, ion/liquid transport, ENaC, Na^+^/K^+^-ATPase, AQP5, lung homeostasis

## Abstract

Alveolar ion and fluid absorption is essential for lung homeostasis in healthy conditions as well as for the resorption of lung edema, a key feature of acute respiratory distress syndrome. Liquid absorption is driven by active transepithelial sodium transport, through apical ENaC Na^+^ channels and basolateral Na^+^/K^+^-ATPase. Our previous work unveiled that KvLQT1 K^+^ channels also participate in the control of Na^+^/liquid absorption in alveolar epithelial cells. Our aim was to further investigate the function of KvLQT1 channels and their interplay with other channels/transporters involved in ion/liquid transport *in vivo* using adult wild-type (WT) and KvLQT1 knock-out (KO) mice under physiological conditions and after thiourea-induced lung edema. A slight but significant increase in water lung content (WLC) was observed in naïve KvLQT1-KO mice, relative to WT littermates, whereas lung function was generally preserved and histological structure unaltered. Following thiourea-induced lung edema, KvLQT1-KO did not worsen WLC or lung function. Similarly, lung edema was not aggravated by the administration of a KvLQT1 inhibitor (chromanol). However, KvLQT1 activation (R-L3) significantly reduced WLC in thiourea-challenged WT mice. The benefits of R-L3 were prevented in KO or chromanol-treated WT mice. Furthermore, R-L3 treatment had no effect on thiourea-induced endothelial barrier alteration but restored or enhanced the levels of epithelial alveolar AQP5, Na^+^/K^+^-ATPase, and ENaC expressions. Altogether, the results indicate the benefits of KvLQT1 activation in the resolution of lung edema, probably through the observed up-regulation of epithelial alveolar channels/transporters involved in ion/water transport.

## 1 Introduction

The epithelial tissue lining alveoli is essential to lung homeostasis and function. The alveolar epithelium is composed of alveolar type I (ATI) cells, responsible for gas exchange, and alveolar type II (ATII) cells, which produce and secrete the surfactant, crucial for alveolar stability and lung compliance. In addition, ATII cells are regarded as progenitor cells for lung self-regeneration and repair after injury (Barkauskas et al., 2013). ATI and ATII cells also play a critical role in the control of fluids at the surface of alveoli. Indeed, fluid absorption, through the paracellular route or *via* aquaporins (AQP) across ATI and ATII cells, is essential to maintain alveolar air spaces virtually free of liquid, necessary for effective gas exchange. Liquid clearance is essentially driven by sodium (Na^+^) absorption, which depends on passive entry, mainly through apical ENaC channels, and then on active Na^+^ exit by the basolateral Na^+^/K^+^-ATPase. Potassium (K^+^) ion recycling, *via* basolateral K^+^ channels, is also crucial to maintain the driving force for Na^+^ absorption (Basset et al., 1987).

The importance of Na^+^ transport, through ENaC channels, in alveolar fluid clearance has been demonstrated *in vitro* as well as in various animal models, in physiological conditions, and after acute lung injury (Basset et al., 1987; O’Brodovich et al., 1990; Smedira et al., 1991; Hummler et al., 1996; Fang et al., 2004; Li and Folkesson, 2006; Matthay, 2014). Notably, Hummler et al*.*,1996 reported that α-ENaC knock-out (KO) mice were unable to clear alveolar fluid at birth and rapidly died from respiratory distress (Hummler et al., 1996). Although partial transgenic expression of α-ENaC in αENaC(−/−)Tg + mice rescued the lethal lung phenotype after birth, the remaining impairment in Na^+^ transport was associated with a lower resolution rate of acute pulmonary edema after thiourea-induced lung injury (Egli et al., 2004; Aubin Vega et al., 2021). Several experimental studies also demonstrated the role of the Na^+^/K^+^-ATPase in alveolar edema clearance (Berthiaume et al., 1987; Olivera et al., 1994; Saldías et al., 1998). Furthermore, previous work highlighted the importance of K^+^ channel function in lung ion and fluid absorption. Indeed, it has been reported that KCa3.1 and K_ATP_ channels play a role in Na^+^ and K^+^ transport pathways and alveolar fluid clearance (Han et al., 2010). Our previous *in vitro* studies also revealed that KvLQT1 and K_ATP_ channels, which contribute to the major part of basolateral K^+^ currents across primary rat alveolar epithelial cells, participate in the control of Na^+^ transport, through the regulation of ENaC mRNA and protein expression (Leroy et al., 2004, 2006; Bardou et al., 2012). Furthermore, we demonstrated that pharmacological activation of KvLQT1 or K_ATP_ channels secondarily enhanced alveolar fluid clearance through the alveolar epithelium *in vitro* (Leroy et al., 2006; Bardou et al., 2012). In resected human lungs, (Sakuma et al.,1998) also reported that a K_ATP_ channel opener favored alveolar liquid absorption, by a process secondarily mediated by amiloride-sensitive ENaC channels (Sakuma et al., 1998). However, to the best of our knowledge, the function of KvLQT1 channels in lung fluid clearance has never been studied before *in vivo*.

Therefore, the aim of our study was to investigate the function of KvLQT1 channels *in vivo*, using complementary molecular and pharmacological approaches, i.e., KO mice with a targeted mutation in the gene coding for the α-subunit (KCNQ1) of the KvLQT1 channel and lung treatments with pharmacological KvLQT1 modulators. KvLQT1 function was studied in physiological conditions, and then, in a model of pulmonary edema induced by a thiourea challenge to assess the impact of KvLQT1 modulation on fluid clearance. This well-established, long-standing model is characterized by the development of lung edema, within both interstitial and alveolar spaces, peaking at ∼4 h after thiourea injection (Hesse and Loosli, 1949; Cunningham and Hurley, 1972; Havill and Gee, 1987; Egli et al., 2004), which initial rapid removal has been shown to be preferentially driven through alveolar reabsorption (Havill and Gee, 1987). Our results indicated that KvLQT1 activation favors the resolution of lung edema at 4 h, probably through the observed up-regulation in alveolar epithelial ENaC channels, AQP5, and Na^+^/K^+^-ATPase expression.

## 2 Materials and methods

### 2.1 Animals and ethical statements

Mice with a targeted disruption in the *kcnq1* gene (constitutive *kcnq1* ^−/−^ knock-out (KO) mice), coding for the KCNQ1 protein (α-subunit of the KvLQT1 K^+^ channel) was originally generated by insertion of a neomycin cassette into exon two by Dr. K. Pfeifer’s group (Laboratory of Mammalian Genes and Development, NICHD/National Institutes of Health, Bethesda, United States), as previously described (Casimiro et al., 2001). The initial breeding pairs (on a C57BL/6J background) were kindly given by Dr. K. Pfeifer. The mouse colony was then maintained by breeding heterozygous mice (male *kcnq1* ^+/-^ x female *kcnq1* ^+/-^) at the Centre de Recherche du Centre Hospitalier de l'Université de Montréal (CRCHUM) animal care facility and backcrossed with C57BL/6J wild-type mice (purchased from Jackson Laboratory) every 10 generations. Animals were maintained in a controlled environment with *ad libitum* access to water and food (2018 Teklad global 18% protein rodent diets, Envigo, United States). Pups were ear punched for genotyping by PCR (using G-KOF (5′-CCA GGA GTG GGT GGT TCT AC -3′), G-KONF (5′-CGC TTC CTC GTG CTT TAC G-3′) and G-KOR (5′-GCC AGC ACT AAA GAT CTT GC-3′) primers (Integrated DNA Technologies, United States) amplifying 240 and 370-bp products, for WT and mutant alleles) at weaning, and designed WT and KO mice. All procedures involving the use of animals were approved by the Institutional Committee for the Protection of Animals (CIPA) at the CRCHUM, in agreement with Canadian Council for Animal Care (CCAC) guidelines.

### 2.2 *In vivo* experimental design

*In vivo* experiments were performed on 6–10 weeks old mice, randomly divided into the experimental groups described below (matched for weight and sex). WT and KO mice were first compared under basal physiological conditions and then after thiourea-induced acute lung edema. Thiourea (TU, 5 mg/kg, Sigma) was injected intraperitoneally (i.p., 150 µl); the control group received the same volume of PBS (i.p.) (i.e., four experimental groups: WT/PBS, KO/PBS, WT/TU, and KO/TU).

The effect of pharmacological treatments with KvLQT1 modulators was also assessed. Briefly, mice received the KvLQT1 activator R-L3 (4 μM, 1/1000 DMSO (vehicle, (veh)) in PBS, Tocris Bioscience, UK), the KvLQT1 inhibitor chromanol (Chrom, 20 μM, 1/1000 DMSO (veh) in PBS, Tocris Bioscience United States), a combination of chromanol and R-L3 (R + C) or PBS (PBS + veh (1/1000 DMSO in PBS, Sigma)) by intranasal instillation (i.n., 50 μl), 1 h before the treatment with thiourea (TU, i.p.) or PBS (PBS, i.p.). Groups (i.n./i.p.): PBS/PBS, Chrom/PBS, R-L3/PBS, PBS/TU, Chrom/TU, R-L3/TU and/or R + C/TU are indicated in each figure legends, as appropriate. 4 h after injection of thiourea (or PBS, i.p.), mice were euthanized with an overdose of pentobarbital (i.p.), and lungs were removed to perform water lung content (WLC) assays, histology/immunofluorescence (IF) analyses or isolation of primary airway or alveolar epithelial cells (see below).

### 2.3 Water lung content assay

After euthanasia, the inferior vena cava was severed; the lungs were removed and directly weighed (wet weight, W). Lungs were heated to 95°C for 24 h to measure the dry weight (D) and then to calculate the water lung content using the formula (Kabir et al., 2002):

$$WLC\;\left(mg/g\right)=\frac{wet\;weight-dry\;weight}{mice\;weight}$$

### 2.4 Evans blue extravasation assay

The Evans Blue extravasation assay, a commonly used technique for pulmonary permeability evaluation after endothelial damage, was performed on thiourea-challenged mice. Briefly, animals were treated with PBS or R-L3 (4 μM, i.n.) before thiourea (5 mg/kg, i.p.) administration and finally, three and a half hour later, a solution of Evans Blue (30 mg/kg, Sigma) was injected by the tail vein (i.v.). After 30 min of blood circulation, mice were euthanized; lungs were perfused with PBS-EDTA (5 mM, 1 mL) *via* the pulmonary artery, collected, and washed in PBS baths. Lungs were then minced with scissors and incubated with formamide (Sigma) for 18 h at 37°C. The homogenate was centrifuged and the luminescence in the supernatant was measured at 620–740 nm, according to a standard curve of Evans Blue dye. Results were corrected for heme pigment with the following formula:

$$E620\;\left(EBD\;corrected\right)=E620-\left(1.426\;x\;E740+0.030\right)$$

### 2.5 Histological analyses and immunostainings (pro-SPC and podoplanin) of cryomatrix-embedded frozen lung tissue sections

To prevent alveolar collapse, a solution of 4% paraformaldehyde (500 μl, Electron microscopy sciences, United States) was administered by intratracheal instillation (i.t.) before lung collection. Tissue samples were incubated in a sucrose gradient (24 h for each gradient of 5%, 10%, and 20%) in a solution of paraformaldehyde (.4%) and embedded in resin (Shandon Cryomatrix, Thermo Fisher Scientific). After cryosections (5 μm) with a cryostat device (Leica Microsystems, CM1950110111 model), slides were then stained with hematoxylin and eosin (Rapid-Chrome Frozen Sections Staining Kit, ThermoScientific, United States) and scanned with a Versa stand on a Leica^®^ light microscope, before histological analysis.

For pro-SPC and podoplanin immunostainings, tissue sections were fixed with 4% paraformaldehyde, membranes were permeabilized with .01% Triton X-100 and then blocked with a solution of PBS +10% FBS (Saradigm, United States) + 10% BSA (Sigma-Aldrich) for 1 h. Slides were incubated overnight at 4°C with anti-pro-SPC (1:100, #AB3786 Millipore, United States) or anti-podoplanin (1:100, #Ab109059, Abcam) rabbit polyclonal antibodies. The day after, tissues were blocked again and incubated with Alexa Fluor™ 568 conjugated donkey anti-rabbit secondary antibodies (1:200, Life Technologies, United States) for 1 h followed by a DAPI staining (1:1000, Sigma) before mounting with Prolong^®^ Gold (Invitrogen, Thermo Fisher Scientific). Pictures were taken with an Exiqua camera (QImaging, Canada) under an inverted fluorescence microscope (Olympus, Canada) at 200x (NA = .75) and analyzed with ICY software. Our analysis protocol allows a quantitative measurement of the intensity (in pixels) of the specific signal of each region of interest (ROI) (stained with each specific primary antibody), normalized to the total number of DAPI-positive cells (more than 10,000 cells have been analyzed for each staining). This ICY protocol also enables adjustment of the parameters of ROI detection to eliminate any potential background signal. However, our control assays (Supplementary Figure S1) showed an absence of background, confirming the specificity of all primary and secondary antibodies used in our assays.

### 2.6 AQP5 immunostaining on formalin-fixed, paraffin-embedded lung tissue sections

After mice euthanasia, an i.t. instillation of formalin (500 μl, Chaptec, Canada) was made before lung collection. Tissue was fixed in 10% neutral buffered formalin, then in paraformaldehyde 4% (Electron microscopy sciences, United States), dehydrated in a series of ethanol solutions of increasing concentrations, cleared in xylene (Chaptec), and embedded in paraffin (Leica Biosystems, United States). Tissue sections (5 μm) were deparaffinized in xylene and rehydrated in a series of ethanol solutions of decreasing concentrations. For AQP5 detection by immunostaining, tissue sections were processed for heat-induced antigenic retrieval (with citrate buffer, pH 3.45) and then blocked with a solution of PBS +10% FBS (Saradigm, United States) + 10% BSA (Sigma-Aldrich) + .01% Triton X-100 (Amersham Biosciences, Sweden) for 1 h. Slides were then incubated overnight at 4°C with an anti-AQP5 rabbit polyclonal antibody (1:100, #AQP-005, Alomone Labs, Israël). The day after, they were blocked again and incubated with an Alexa Fluor™ 568 conjugated donkey anti-rabbit secondary antibody (1:200, Life Technologies, United States) for 1 h followed by a DAPI staining (1:1000, Sigma) before mounting with Prolong^®^ Gold (Invitrogen, Thermo Fisher Scientific). Pictures were taken with an Exiqua camera (QImaging, Canada) under an inverted fluorescence microscope (Olympus, Canada) at 200x (NA = .75) and analyzed with ICY Software (see Section 2.5).

### 2.7 Lung function measurements

The mechanical properties of the respiratory system were assessed in live WT and KO mice, challenged, or not, with thiourea and treated or not with the KvLQT1 activator, R-L3, using the flexiVent FX system (SCIREQ, Montreal, QC, Canada), as previously described (Robichaud et al., 2017). Briefly, mice received an administration of xylazine hydrochloride (12 mg/mL, i.p) 5 min before being deeply anesthetized with sodium pentobarbital (70 mg/kg, i.p.). They were then tracheotomized, cannulated (18-gauge metal cannula having a typical resistance of .22 cmH_2_O.s/mL), and connected to a computer-controlled small animal ventilator for mechanical ventilation. The following settings were used: a tidal volume of 10 mL/kg, frequency of 150 breaths/min, inspiratory to expiratory ratio (I:E) of 2:3, and positive end-expiratory pressure (PEEP) of three cmH_2_O. Data acquisitions started after two deep lung inflations to 30 cmH_2_O, to open closed lung areas as well as to standardize lung volumes, and following observation of a stable ventilation pattern without spontaneous effort. If needed, an additional dose (.25–.5 mg/kg, i.p.) of the anesthetic agent was administered and approximately 2 min of default ventilation was applied to allow for the drug effect to be reached. Four consecutive and different measurement perturbations were performed as one cycle, and the cycle was repeated until three acceptable measurements were recorded. The entire measurement sequence lasted on average 4–5 min per subject and lung function was assessed either under basal physiological conditions or 4 h after thiourea-induced lung edema. More specifically, pressure-volume (PV) curves were constructed by inflating the lungs by increments of pressure from PEEP up to 30 cm H_2_O and then deflating in a similar manner. Pressure and volume signals were recorded at each increment following a brief plateau (1 s) and used to construct partial PV curves. The deflation limbs were analyzed as previously described (Robichaud et al., 2017) and used for the determination of quasi-static compliance (*C*_*st*_). The area between the inflation and deflation limb (Area) was also calculated and the work-of-breathing was determined from the area under the inflation curve normalized to the volume at maximal pressure and expressed per liter to reflect a normalized inspiratory work of breathing (WOBn) from a large amplitude maneuver (Phillips et al., 2012; Gremlich et al., 2020; Robichaud et al., 2021). The mechanical properties of the respiratory system were also determined, using both single (SnapShot-150) and broadband (Quick Prime-3) forced oscillation perturbations. The overall resistance (*R*_*rs*_), elastance (*E*_*rs*_) and compliance (*C*_*rs*_) of the respiratory system were obtained, as previously described (Robichaud et al., 2017), from the fitting of the classic single compartment model to the experimental signals of a single forced oscillation measurement. Similarly, the constant-phase model (Hantos et al., 1992) was fit to the respiratory input impedance data from the broadband forced oscillation perturbation to describe the mechanical properties of the subject’s respiratory system using parameters such as the Newtonian resistance (R_N_) which is dominated by the airway resistance as well as tissue elastance (*H*) and tissue damping (*G*) to respectively express the stiffness of the respiratory tissues and the tissue resistance which contains a contribution from the small peripheral airways. Finally, an assessment of the subject’s inspiratory capacity was obtained from the deep lung inflation maneuver to 30 cm H_2_O, as previously described (Robichaud et al., 2017). At the end of the experiment, averages of the collected datasets were calculated, first for each subject and parameter and then for each experimental group.

### 2.8 Isolation of mouse airway epithelial cells

Mouse airway epithelial cells were isolated from non-treated WT and KO mice. After dissection, the trachea was cut lengthwise and incubated for the night at 4°C in minimal essential medium (MEM, Life Technologies) supplemented with 7.5% NaHCO_3_ (Sigma-Aldrich), 2 mM L-glutamine (Invitrogen, Ontario, Canada), 10 mM HEPES (Thermo-Fisher Scientific Inc., #SH3023701), .05 mg/mL gentamycin (Life Technologies), 50 U/mL penicillin-streptomycin (ThermoScientific), and containing a mix of .1% Protease E (from *Streptomyces griseus*; Sigma-Aldrich) and 10 μg/ml DNAse (Deoxyribonuclease I from bovine pancreas; Roche, United States). The cells were then gently scraped off the cartilaginous structure and the activity of protease/DNAse was neutralized by adding FBS. The collected cell suspension was then centrifuged, counted, and seeded into Transwell^®^ (.4 μm pore size, Corning, United States) coated with Purecol^®^ (Cedarlane, Burlington, Canada). Cells were cultured in CnT-17 medium (CELLnTEC Advanced Cell Systems, Bern, Switzerland) + 20% FBS for 13 days. Then, the medium bathing the apical side of the airway epithelium was removed to create an air-liquid interface. Forty-8 h later, the medium from the basolateral compartment was replaced by complete SAGM™ (LHC basal medium supplemented with the SAGM™ kit, Clonetics, Walkersville, MD) added with 25 ng/mL EGF (Sigma-Aldrich), 100 U/mL of penicillin-streptomycin (ThermoScientific), .07 μg/ml phosphorylethanolamine (Sigma-Aldrich), 1.86 ng/ml ethanolamine (Sigma-Aldrich), .05 nM retinoic acid (Sigma-Aldrich), electrolytes and trace elements (kindly provided by Dr. Scott Randell, University of North Carolina) for 2 days, before electrophysiological experiments in Ussing chambers.

### 2.9 Measurement of short-circuit currents in Ussing chambers

Short-circuit currents (*I*_sc_) were measured through polarized cultures of airway epithelial cells (isolated from WT and KO mice) grown on permeant filters at the air-liquid interface. After stabilization of the short-circuit current, the total basolateral K^+^ current was determined after permeabilization of the apical membrane with amphotericin B (7.5 µM, Sigma-Aldrich) and establishment of an apical-to-basolateral K^+^ gradient. The apical side was bathed with a high K^+^ physiological solution containing: 81 mM NaCl, 65.4 mM KCl, .78 mM NaH_2_PO_4_, .8 mM MgCl_2_, 1.8 mM CaCl_2_, 5 mM glucose, and 15 mM HEPES, pH 7.4, whereas for the basolateral side, 60 mM of KCl was replaced by an equivalent amount of *N*-methyl-D-glucamine chloride. Therefore, all ion concentrations, except K^+^, were symmetrical in solutions bathing both sides of the membrane, meaning that only K^+^ ions can be conducted through the basolateral membrane. In these experimental conditions, the difference between total K^+^ basolateral currents in cell cultures from WT and KO mice corresponds to the current driven by KvLQT1 channels. After stabilization of the current, 20 μM chromanol was added to the basolateral medium, to determine the chromanol-sensitive basolateral K^+^ current through KvLQT1 channels.

### 2.10 Isolation of alveolar type II (ATII) epithelial cells

ATII cells were isolated from WT mouse lungs in the control condition and after thiourea-induced acute lung edema treated or not with R-L3. After collection, the lungs were washed with a physiological solution to remove excess blood cells and alveolar macrophages. Then, the lungs were digested within an elastase solution (30 U/mice, Worthington Biochemical, Lakewood, N.J. United States, 30–45 min), minced, and the resulting cell suspension was filtered. Alveolar epithelial cells were purified using a differential adherence technique (Dobbs et al., 1986), which enhances the purity of the ATII cell pool by up to 86% (Brochiero et al., 2004). Cells were counted and the cell suspension was resuspended in PBS to reach a density of 80,000 cells (in 200 μl PBS/slide) after cytocentrifugation with Cytospin 4 (Thermo Fisher, UK) for immediate immunofluorescence assay.

### 2.11 α-ENaC and Na^+^/K^+^-ATPase immunostaining of ATII cells

Cytocentrifuged, freshly isolated ATII cells were fixed with 4% paraformaldehyde, membranes were permeabilized with .01% Triton X-100 (for Na^+^/K^+^-ATPase staining) and then blocked with a solution of PBS +10% FBS (Saradigm, United States) + 10% BSA (Sigma-Aldrich) (before α-ENaC staining) or with a solution of PBS +1% BSA + 5% goat serum (Life Technologies, United States) (before Na^+^/K^+^-ATPase staining). Slides were incubated overnight at 4°C with anti-α-ENaC rabbit polyclonal (1:100, #AB2184369, Invitrogen, United States) or anti-Na^+^/K^+^-ATPase mouse monoclonal (1:100, clone C464.6, Millipore, United States) antibodies. The day after, they were blocked again, incubated with Alexa Fluor™ 568 conjugated donkey anti-rabbit secondary (α-ENaC staining) or Alexa Fluor™ 568 conjugated goat anti-mouse secondary antibodies [(Na^+^/K^+^-ATPase staining), 1:200, Life Technologies, United States] for 1 h, followed by a DAPI staining (1:1000, Sigma) before mounting with Prolong^®^ Gold (Invitrogen, Thermo Fisher Scientific). Pictures were taken with an Exiqua camera (QImaging, Canada) under an inverted fluorescence microscope (Olympus, Canada) at 200x (NA = .75) and analyzed with ICY software (see Section 2.5).

### 2.12 Statistical analyses

The data are presented in dot plot graphs with mean ± standard error of the mean (SEM). Graphs and statistical analyses were performed with GraphPad Prism version eight for Windows (GraphPad Software, San Diego, CA, United States). First, normality tests (Agostino/Pearson) were performed followed by the appropriate statistical tests, as described for each figure legend. Differences were considered significant when *p <* .05.

## 3 Results

**FIGURE 1:**
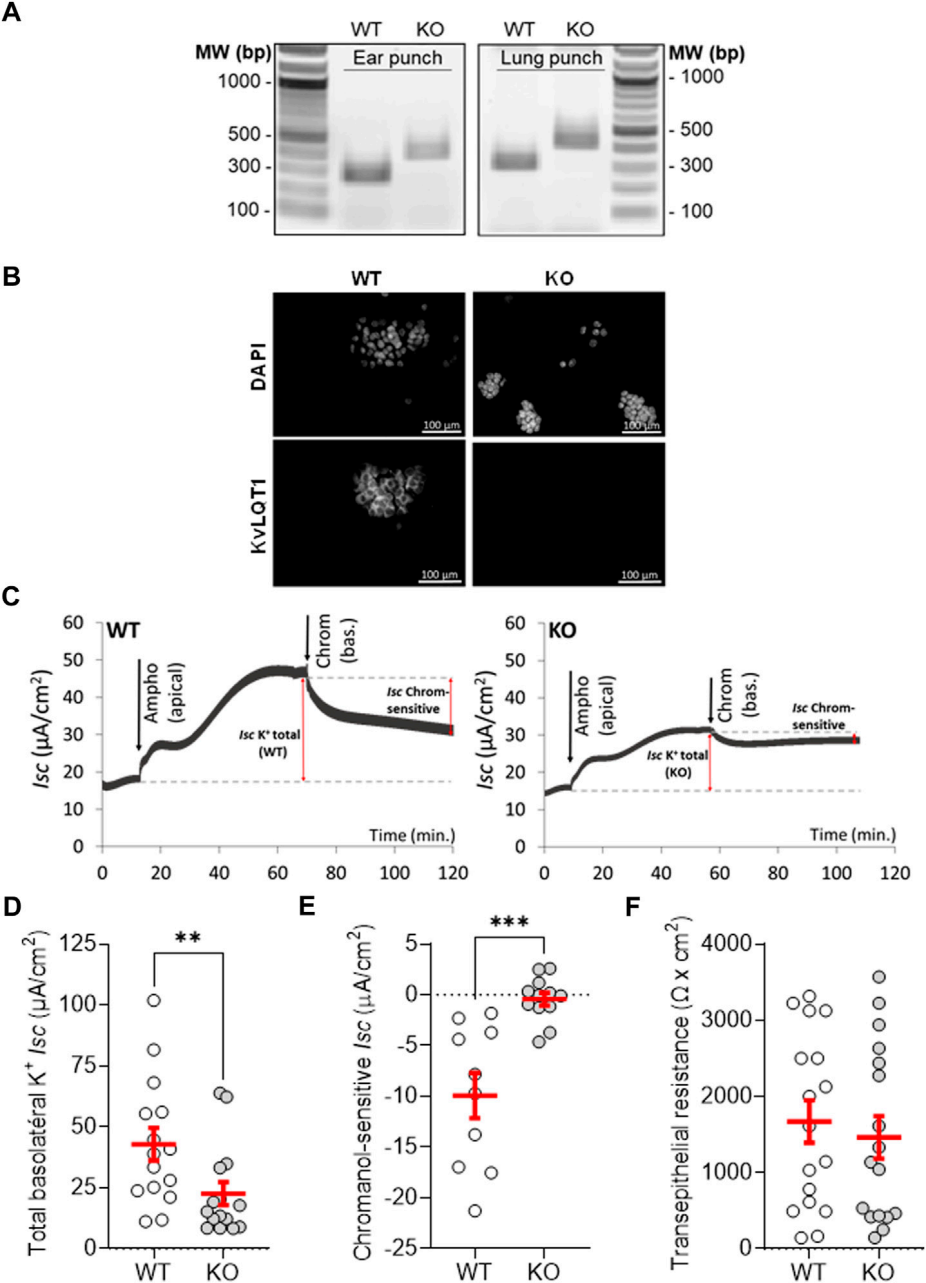
Validation of KvLQT1 extinction in mouse lungs. **(A)** Detection of the 240 and 370-pb products, specific to the WT and mutant alleles, amplified by PCR from genomic DNA of ear punches and lung tissues from WT and KO mice. Representative immunofluorescence images of KvLQT1 channel protein staining performed on freshly isolated mouse airway epithelial cells **(B)**, n = 4, Magnification: × 200 Scale: 100 µm). **(C)** Representative short-circuit current traces, measured in Ussing chamber, of primary cultures of airway epithelial cells, isolated from WT (left panel) and KO mice (right panel). After current stabilization, 7.5 µM amphotericin B was added and an apical-to-basolateral K^+^ gradient was established, before addition of 20 µM chromanol (at the basolateral side). Total basolateral **(D)** and chromanol-sensitive **(E)** K^+^ currents (*I*_*sc*_ μA/cm^2^) as well as transepithelial resistance F (Ωxcm^2^) of WT and KvLQT1-KO cell cultures (n = 10–17). Values are means ± SEM. Non-parametric Mann-Whitney t-test (Agostino/Pearson normality test: negative, panels **(D, E)** and unpaired t-test (Agostino/Pearson normality test: positive, panel **(F)**. **p < .01, ***p < .001 vs*.* WT mice.

Effective *kcnq1* extinction in lung tissues of constitutive *kcnq1* ^*−/−*^ (KO) mice was first validated. As expected, amplification by PCR of genomic DNA extracted from ear and lung tissues of WT and KO mice generated 240 and 370-bp products specific to the WT and mutant alleles (Casimiro et al., 2001), respectively (Figure 1A). Moreover, immunofluorescence assays confirmed KvLQT1 protein extinction in airway epithelial cells collected from KO mice, compared to WT (Figure 1B).

Short-circuit current measurements in Ussing chamber (see representative traces in Figure 1C) showed that the total basolateral K^+^ current in cell cultures from WT mice (42.8 ± 6.7 µA/cm^2^, Figure 1D, WT) was significantly higher than in cultures from KO mice (22.6 ± 4.6 μA/cm^2^, Figure 1D, KO), i.e. a difference of 20.2 µA/cm^2^, corresponding to the current driven by KvLQT1 channels (47% of the total basolateral currents). We also showed that chromanol-sensitive KvLQT1 currents were abolished (-.4 ± .7 μA/cm^2^) through primary cultures of airway epithelial cells, collected from KO mice (Figure 1E). It has to be noted that transepithelial resistances of WT and KO cell cultures were similar (Figure 1F).

**FIGURE 2:**
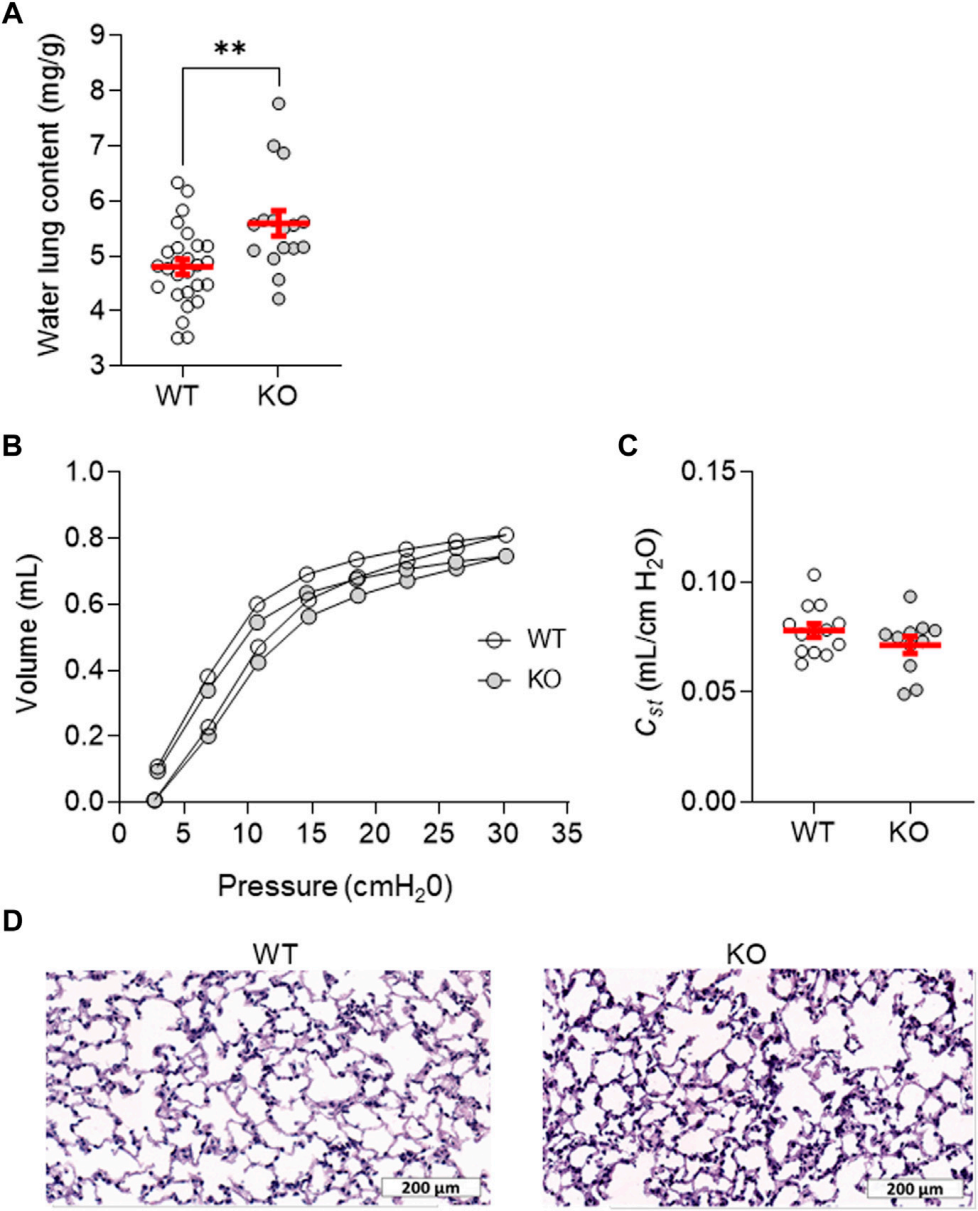
Lung phenotype assessment of the KvLQT1-KO mouse model. **(A)** Water lung content (mg/g) from control (non-treated) WT and KO mice (n = 16–27). Mean pressure-volume loops, **(B)** and C_st_ (quasi-static compliance at 5 cmH_2_O, **(C)** were measured with flexiVent among control (non-treated) WT and KO mice (n = 11–13). Representative images of histological sections from WT and KO mouse lungs stained with hematoxylin-eosin **(D)**, Scale: 200 μm). Values are presented as means ± SEM. Unpaired t-test (Agostino/Pearson normality test: positive) was practiced for panel **(A-C)**. **p < .01 vs*.* WT mice.

The impact of KvLQT1-KO has already been studied in several organs (Casimiro et al., 2001; Knollmann et al., 2004; Boini et al., 2009; Song et al., 2009; Wang et al., 2009), but, to the best of our knowledge, the lung phenotype has not been defined yet. The observed reduction (47%, *p <* .01) in total basolateral K^+^ currents in KO mice indicated the large contribution of KvLQT1 channels (Figure 1D), while remaining K^+^ currents can be conducted by other classes of K^+^ channels also expressed in the lung (Bardou et al., 2009). KvLQT1-KO was associated with a small but statistically significant rise in water lung content, compared to WT (Figure 2A). However, lung function analyses showed that the PV curves (Figure 2B) constructed with the flexiVent system, as well as static compliance (C_st_, Figure 2C) from the two experimental groups were similar. Slight but significant changes were measured in overall respiratory system resistance (*Rrs*), elastance (*Ers*), and compliance (*Crs*) as well as tissue damping (*G*), related to alterations of the peripheral tissues. No difference was observed for the other respiratory mechanic parameters measured, including airway resistance (R_N_) (Supplementary Figure S2). Also, no histological differences were noticeable between lungs from WT and KO mice (Figure 2D). These results indicate that the KvLQT1 deletion did not elicit major functional and structural impairments under basal physiological conditions.

**FIGURE 3:**
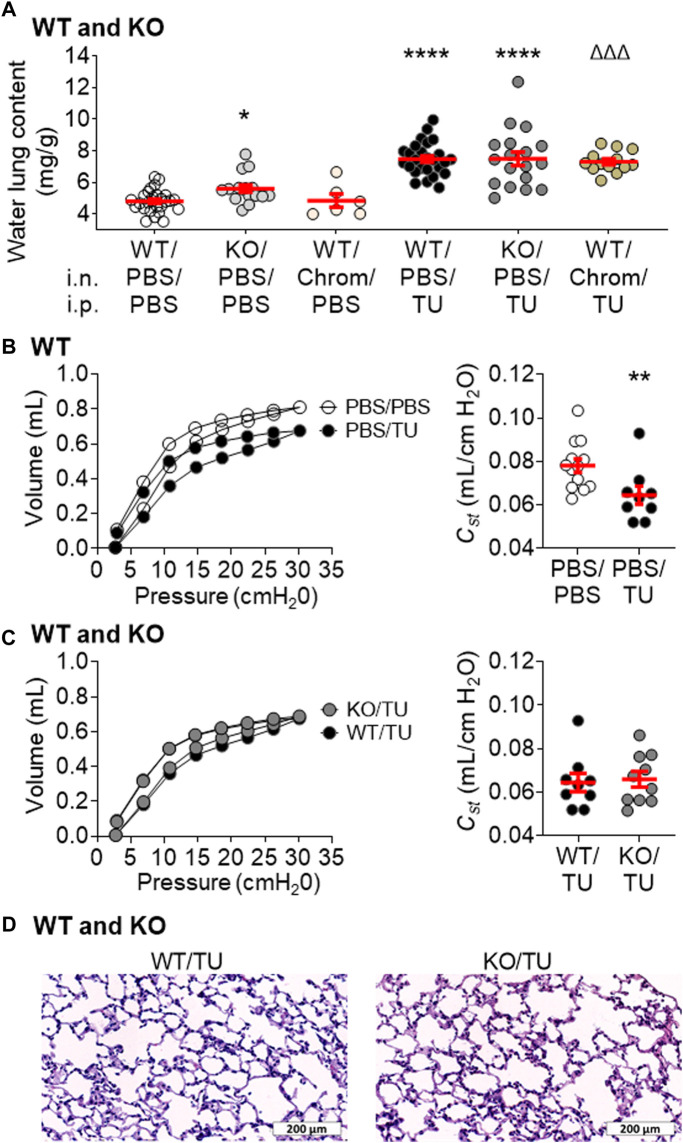
Impact of KvLQT1 extinction and pharmacological inhibition on thiourea-induced acute lung edema and function in mice. **(A)** WT mice were pretreated with PBS (i.n.) or with the KvLQT1 inhibitor chromanol (Chrom, 20 μM, i.n.) 1 h before i.p. injection (150 μl) of PBS (control condition) or thiourea (TU, 5 mg/kg). KO mice also received PBS (i.n) prior to PBS or thiourea injection. 4 h later, lungs were collected for measurement of the water lung content within the six experimental groups in WT mice (i.n/i.p): PBS/PBS, Chrom/PBS, PBS/TU, and Chrom/TU and KO mice: PBS/PBS and PBS/TU, n = 6–27. Mean pressure-volume loops and *Cst* were also measured in separate WT control (PBS/PBS) and thiourea-treated (PBS/TU) mice **(B)**, n = 9–13 with the flexiVent system as well as in WT and KO thiourea-treated mice **(C)**, WT/TU vs. KO/TU, n = 9–10. Representative images of histological sections from WT and KO thiourea-treated mouse lungs stained with hematoxylin-eosin **(D)**, Scale: 200 μm. Values are presented as means ± SEM. One-way ANOVA and Bonferroni’s multiple comparisons test were practiced (normality Agostino/Pearson test: positive) and One-way ANOVA non-parametric comparison test (normality Agostino/Pearson test: negative) were applied to panel **(A)**. Non-parametric Mann-Whitney t-test (Agostino/Pearson normality test: negative) was practiced for panel **(B, C).** *p < .05, **p < .01, ****p < .0001 vs*.* PBS/PBS, ^ΔΔΔ^ *p* < .001 vs*.* Chrom/PBS.

We then evaluated the role of KvLQT1 channels using a well-established acute model of thiourea-induced lung edema, peaking at 4 h (Hesse and Loosli, 1949; Cunningham and Hurley, 1972; Havill and Gee, 1987; Egli et al., 2004). As expected, increased water lung content was observed in WT mice challenged with thiourea (WT/PBS/TU, Figure 3A). In KO mice, similar levels of lung edema were measured indicating a comparable response to thiourea (WT/PBS/TU vs KO/PBS/TU groups, Figure 3A). The impact of KvLQT1 pharmacological inhibition in thiourea-challenged mice was also assessed. Chromanol administration to the lungs (by intranasal instillation) did not worsen the edema index of thiourea-challenged WT mice (WT/PBS/TU vs. WT/Chrom/TU groups, Figure 3A).

In addition to the lung edema, a shift in the shape of the PV curve (Figure 3B, left panel), as reflected by a slight but statistically significant decrease in the static compliance (*C*_*st*_; Figure 3B, right panel) was observed after the thiourea challenge in WT mice. As shown in Supplementary Figure S3, other parameters (*Ers, Crs, A,* IC, and *H*) are also modified after thiourea-induced lung edema (PBS/TU).

Consistent with the water lung content results, no worsening of the lung function parameters (Figure 3C; Supplementary Figure S4) was observed in KO animals which exhibited similar sensitivity to thiourea. Moreover, similar histological features were seen in WT and KO thiourea mice (Figure 3D).

**FIGURE 4:**
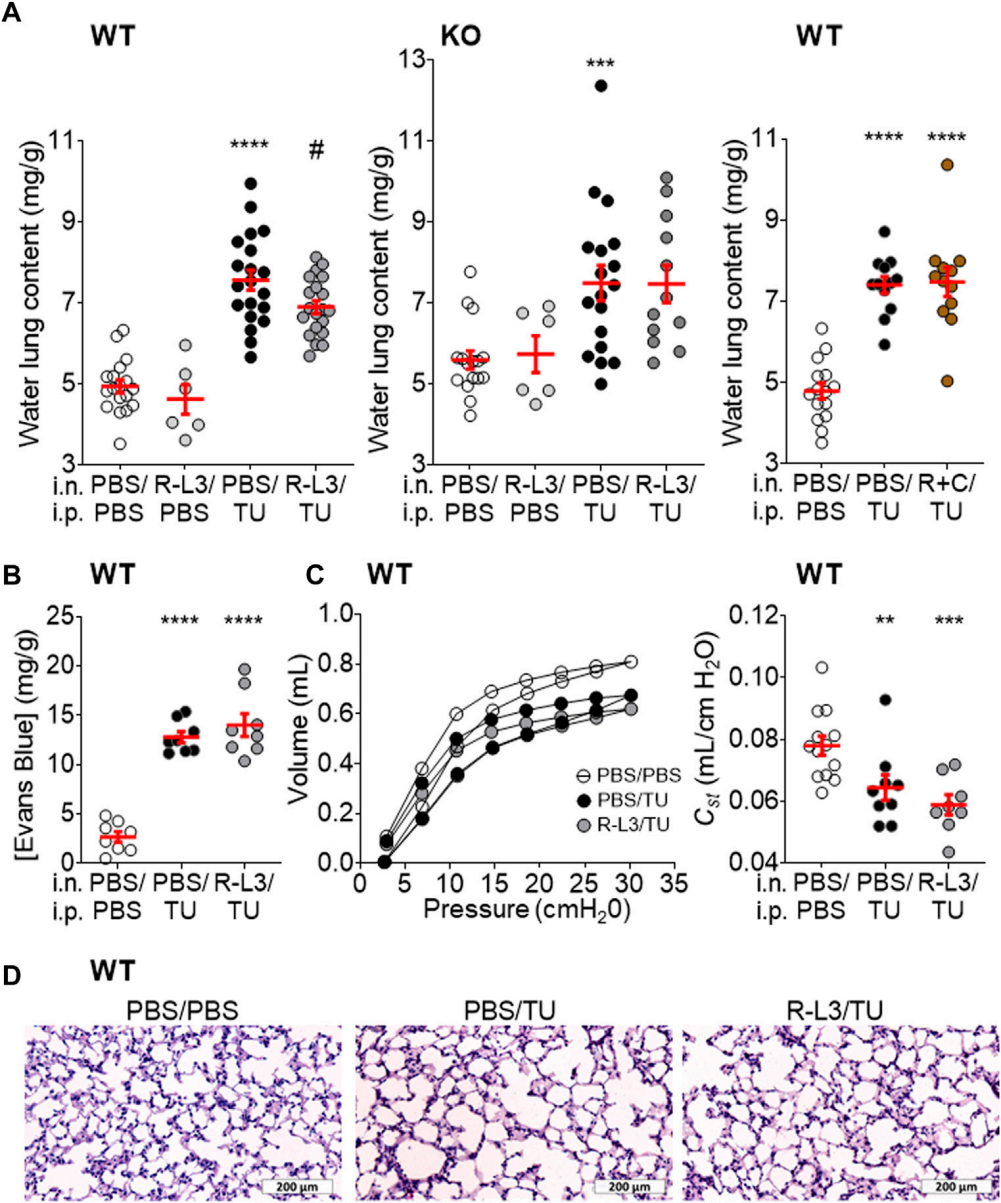
Beneficial impact of KvLQT1 activation on thiourea-induced acute lung edema in WT mice. **(A)** After treatment with the pharmacological KvLQT1 activator R-L3 (4 μM, i.n.) or PBS (i.n.), a group of WT (left panel, n = 6–20) or KO (middle panel, n = 6–18) mice were challenged with thiourea (TU, 5 mg/kg, i.p.) or PBS (i.p.). 4 h after, lungs were collected for measurement of the water lung content within the four experimental groups (i.n/i.p: PBS/PBS, R-L3/PBS, PBS/TU, and R-L3/TU). Also, WT mice were treated with a combination of R-L3+chromanol (R + C, 4 μM and 20 µM respectively, i.n) or PBS before the challenge with thiourea (TU, 5 mg/kg, i.p.) or PBS (i.p.) and the water lung content was compared among the three experimental groups (i.n/i.p: PBS/PBS, PBS/TU, and R + C/TU, n = 12–15) (right panel). Endothelial permeability **(B),** n = 8, assessed by Evans blue (20 mg/kg, 100 μL, i.v.) extravasation from the circulation to the lungs, was measured in the control condition (i.n./i.p.: PBS/PBS) and after the thiourea challenge (i.p.: TU, 5 mg/kg) in WT mice treated with the KvLQT1 activator R-L3 (i.n/i.p.: R-L3/TU) or not (PBS/TU). Mean pressure-volume loops **(C)**, middle panel and *Cst* **(C)**, right panel (n = 8–13) in WT control animals (PBS/PBS) and mice treated with R-L3 (i.n/i.p.: R-L3/TU) or not (PBS/TU) were measured with the flexiVent system. Representative images of histological sections from WT thiourea mouse lungs treated or not with the KvLQT1 activator stained with hematoxylin-eosin **(D)**, Scale: 200 μm). Values are presented as means ± SEM. One-way ANOVA and Bonferroni’s multiple comparisons test were practiced (normality Agostino/Pearson test: positive) for panels **(A–C)**. **p < .01 ***p < .001 ****p < .0001 vs*.* PBS/PBS and ^#^ *p* < .05 vs*.* PBS/TU.

Interestingly, the pharmacological activation of KvLQT1 with R-L3 induced a statistically significant reduction of the water lung content in WT mice treated with thiourea (R-L3/TU mice, compared to the PBS/TU group, Figure 4A, left panel). This beneficial effect of R-L3 was abolished in KvLQT1-KO mice (R-L3/TU KO mice, Figure 4A, middle panel) as well as in WT mice co-treated with the KvLQT1 inhibitor chromanol (R-L3+Chrom/TU (R + C/TU) group, Figure 4A, right panel), thus confirming that the observed decrease in lung edema in R-L3-treated WT mice (R-L3/TU group) is mediated by KvLQT1 channels.

Exposure to thiourea is associated with alveolar capillary injury and a secondary increase in vascular permeability (Giri et al., 1991), as confirmed by the increased Evans Blue dye extravasation from the blood in lung homogenates from thiourea-challenged WT mice (Figure 4B). We thus tested the hypothesis that the beneficial effect of the KvLQT1 activator R-L3 on the edema index could be due to endothelial barrier preservation. Nonetheless, the observed increase in endothelial permeability in thiourea-challenged mice was not prevented by the R-L3 treatment (Figure 4B). Moreover, the treatment with R-L3 did not improve the shape of the PV curve, the static compliance (*C*_*st*,_ Figure 4C), and other respiratory mechanic parameters at the dose tested (Supplementary Figure S3). Hematoxylin-eosin staining of lung sections from WT mice after thiourea-induced lung edema, treated (R-L3/TU) or not (PBS/TU) with R-L3 (Figure 4D) did not reveal any major changes in alveolar epithelial structure.

**FIGURE 5:**
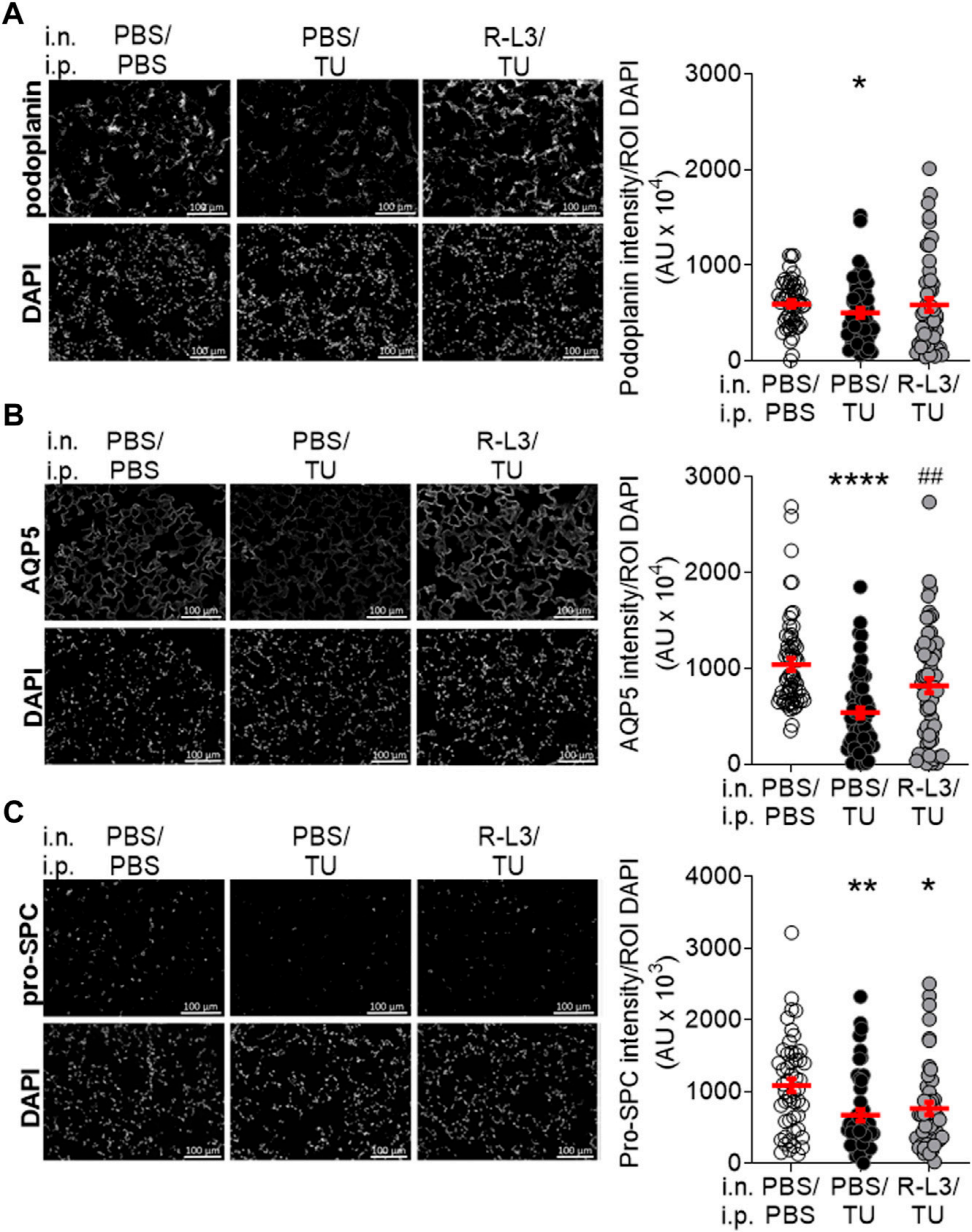
Impact of KvLQT1 activation on alveolar type I (AT1) and II (ATII) epithelial cell markers in thiourea-challenged WT mice. Representative immunofluorescence images (left panels) of lung sections (5 μm, Scale: 100 μm) from WT control mice (PBS/PBS), and WT mice challenged with thiourea (TU, 5 mg/kg i.p., PBS/TU) and treated or not (PBS) with the KvLQT1 activator R-L3 (R-L3/TU) stained with the ATI markers podoplanin **(A)**, n = 50 images, AQP5 **(B)**, n = 59–60 or ATII marker pro-SPC **(C)**, n = 48–51). Nuclei were stained by DAPI. Quantification (right panels) of podoplanin, AQP5, and pro-SPC marker intensities was made with a protocol exploited by ICY software. Values are presented as means ± SEM. Non-parametric Mann-Whitney t-test (Agostino/Pearson normality test: negative) for panel **(A)**. One-way ANOVA non-parametric comparison test (normality Agostino/Pearson test: negative) for panel **(B**, **C)**. *p < .05, ^**^ *p* < .01 or ****p < .0001 vs*.* PBS/PBS, ^##^ *p* < .01 vs*.* PBS/TU.

**FIGURE 6:**
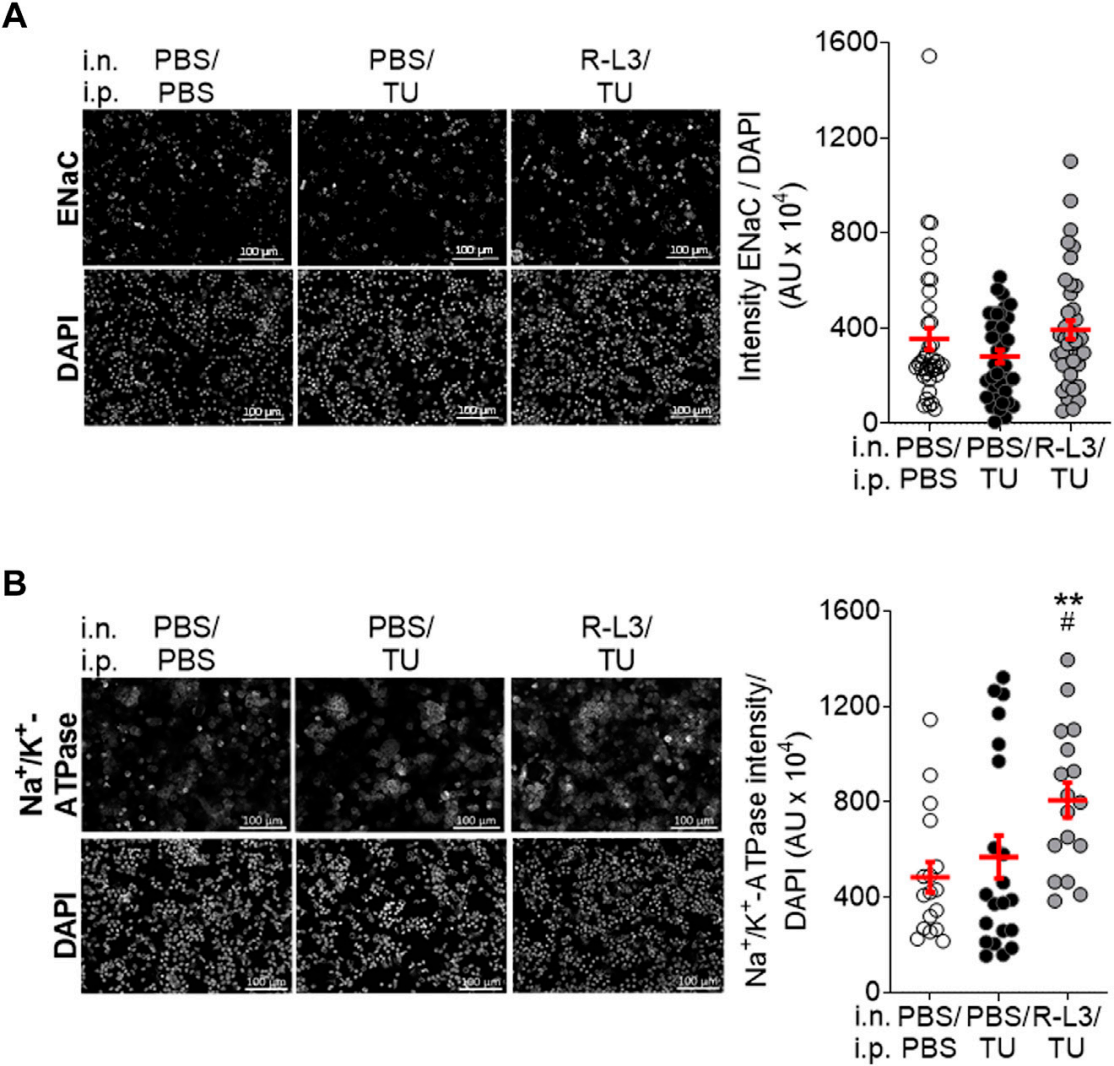
Impact of KvLQT1 activation on α-ENaC and Na^+^/K^+^-ATPase protein expression in alveolar epithelial cells after thiourea-induced lung edema in WT mice. Representative immunofluorescence images (left panels, Scale: 100 μm) of the α-ENaC subunit **(A),** n = 3 experiments, including a pool of 14 mice) and Na^+^/K^+^-ATPase **(B),** n = 3 experiments, including a pool of 14 mice staining performed on ATII cells freshly isolated from lungs from WT control mice (i.n/i.p.: PBS/PBS) and WT mice challenged with thiourea (i.p.: TU, 5 mg/kg) and treated or not (PBS) with the KvLQT1 activator (i.n./i.p.: R-L3/TU and PBS/TU, respectively). Nuclei were stained by DAPI. Quantification (right panels) of marker intensity was made with a protocol exploited by ICY software. Values are presented as means ± SEM. One-way ANOVA non-parametric comparison test (normality Agostino/Pearson test: negative) for panel **(A)**. One-way ANOVA and Bonferroni’s multiple comparisons test were practiced (normality Agostino/Pearson test: positive) for panel **(B)**. **p < .01, vs*.* PBS/PBS and ^#^ *p* < .05 vs. PBS/TU for Na^+^/K^+^-ATPase.

We then postulated that the benefits of KvLQT1 activation on edema resolution could be mediated through an improvement in alveolar epithelial integrity and/or function. To test this hypothesis, we performed immunofluorescence assays with specific markers of alveolar epithelial cells as well as proteins involved in Na^+^, and secondary liquid, absorption (Figures 5,6). Because type I alveolar epithelial (ATI) cells are especially vulnerable to injury, we further assessed the expression of two ATI markers; podoplanin (Figure 5A) and AQP5 (Figure 5B). A statistically significant decrease in podoplanin and AQP5 was observed after the thiourea challenge (PBS/TU). Importantly, KvLQT1 activation with R-L3 (R-L3/TU group) partially restored aquaporin 5 (AQP5) expression; this effect is statistically significant (Figure 5B). We then observed that the staining intensity of the pro-SPC protein (ATII marker, Figure 5C) was significantly reduced in lung sections from thiourea-challenged mice, but treatment with R-L3 failed to reverse this deleterious effect.

Finally, the expression of ENaC channels and Na^+^/K^+^-ATPase was assessed (Figure 6), given their major contribution in Na^+^, and secondarily liquid, absorption through the alveolar epithelium. Immunostainings of primary alveolar epithelial cells, freshly isolated from WT mice, indicated that the intensity of ENaC expression per cell tends to decrease after thiourea-induced lung edema (PBS/TU, compared to PBS/PBS) (Figure 6A), while the R-L3 treatment (R-L3/TU) allows to recover ENaC expression levels to a value slightly above the control (without thiourea) (Figure 6A). Exposure to thiourea (PBS/TU) did not significantly alter the Na^+^/K^+^-ATPase level of intensity (Figure 6B) but significantly reduced the number of Na^+^/K^+^-ATPase marked regions (ROI, not shown). Importantly, KvLQT1 activation with R-L3 (R-L3/TU) upregulated the intensity of Na^+^/K^+^-ATPase expression per DAPI-positive cell (Figure 6B).

## 4 Discussion

The goal of this study was to investigate the function of KvLQT1 channels using *in vivo* complementary molecular and pharmacological approaches in mice, both under physiological conditions as well as following acute lung edema induced by thiourea. We found that the deletion of KvLQT1 channels, which account for approximatively 50% of the total basolateral K^+^ current, was associated, in physiological conditions, with a slight rise in water lung content, whereas neither histological changes nor major alterations in respiratory function were observed. Moreover, neither KvLQT1-KO nor its pharmacological inhibition (with chromanol) worsened the lung edema induced by thiourea. However, lung administration of the KvLQT1 activator R-L3 significantly reduced the thiourea-induced pulmonary edema in WT mice. This beneficial effect was prevented in KO and chromanol-treated WT mice, confirming a KvLQT1-dependent mechanism. R-L3 treatment in WT mice also reversed the thiourea-induced decrease in aquaporin channel AQP5 and ENaC expression and enhanced the Na^+^/K^+^-ATPase levels, which may favor lung edema clearance, as observed.

*Kcnq1* ^*−/−*^ KO mice used in our study were originally generated as a model of Jervell and Lange–Nielsen Syndrome, a disorder featuring profound bilateral deafness and a cardiac phenotype (Casimiro et al., 2001). As expected, we noted that KO mice exhibited head bobbing and intermittent bidirectional circling, a behavior symptomatic of inner ear defects, usually referred to as the shaker/waltzer phenotype. Altered inner ear, cardiac, and thyroid gland phenotypes have already been reported in these KO mice (Casimiro et al., 2001; Tosaka et al., 2003; Knollmann et al., 2004, 2007; Fröhlich et al., 2011). A study on insulin sensitivity also showed an impact of KCNQ1 disruption on glucose uptake in various tissues including lungs (Boini et al., 2009). However, the lung morphological and functional phenotype associated with KvLQT1 disruption has, to the best of our knowledge, never been investigated before, neither in physiological nor pathological conditions.

According to our histological observations of lung sections, the deletion of the KvLQT1 α-subunit KCNQ1 did not cause any morphological changes in the lungs. In agreement with our data, previous work showed that disruption of the KvLQT1 β-subunit (KCNE3) did not alter the morphology of small intestine, colon, tracheal, and lung epithelia, while ion transport properties through the intestinal and tracheal epithelia were dramatically affected (Preston et al., 2010). Structural changes associated with KvLQT1 dysfunction have however been observed in other organs. Indeed, gross morphological anomalies in inner ear structures and severe histological alterations of the gastric mucosa, accompanied by deafness and impaired acid secretion, respectively, have been reported using KCNQ1-KO mice models (Lee et al., 2000; Casimiro et al., 2001).

Functional electrophysiological analyses in Ussing chamber revealed that total basolateral K^+^ currents were reduced by 20.2 μA/cm^2^ (i.e. a 47% decrease) in airway epithelial cells from KO mice, compared to WT (Figure 1D). These data are in agreement with previous reports, including from our laboratory, indicating a major contribution of KvLQT1 channels to the total basolateral K^+^ conductance in murine tracheal and alveolar epithelial cells (Grahammer et al., 2001; Leroy et al., 2004; Trinh et al., 2007) as well as human airway epithelial cell models (Cowley and Linsdell, 2002; Trinh et al., 2008). Our measurements also confirmed that KCNQ1 extinction in KO mice abolished the chromanol-sensitive basolateral K^+^ currents (Figure 1E). We noted however that the amplitude of the K^+^ current sensitive to 20 µM chromanol in WT mice was lower (10 μA/cm^2^) than the decrease (20.2 μA/cm^2^) in total K^+^ current induced by the full extinction of KvLQT1 in KO mice. Higher chromanol concentrations would probably have elicited a larger inhibition, but non-specific effects at a high dose could not have been ruled out.

As illustrated by the individual data reported in Figures 1D, E, the amplitude of the chromanol-sensitive KvLQT1 currents and the impact of KvLQT1 extinction on the total basolateral K^+^ current are variable among animals, suggesting that the relative contribution of KvLQT1 channels may vary in resting conditions. Actually, more than 30 different K^+^ channels, from different classes, have been detected in the respiratory epithelium (Bardou et al., 2009). Although the function of many of these lung K^+^ channels is still unknown, some of them [including KCa and K_ATP_ channels (Cowley and Linsdell, 2002; Bernard et al., 2003; Leroy et al., 2004; Trinh et al., 2008)] may contribute to the remaining K^+^ current observed in KvLQT1-KO cells, in basal conditions.

A small, but significant, increase in basal water lung content was observed in the lungs of KvLQT1-KO mice, compared to WT, under basal physiological conditions (Figure 2A). This observation may be explained, at least in part, by the impact of KvLQT1 disruption on alveolar fluid homeostasis. Indeed, our previous work unveiled that KvLQT1 channel chronic inhibition impaired liquid absorption through primary alveolar cell cultures (Leroy et al., 2006; Bardou et al., 2012). No increase in water lung content was however observed after a short treatment with chromanol in the basal condition in WT mice (Figure 3A, Chrom/PBS). Lung function in KO mice was mostly preserved with only minor changes in some mechanical parameters (*Rrs*, *Ers*, *Crs*, *G*) of the overall respiratory system (Supplementary Figure S2). While the rise in water lung content may contribute to the observed increase in tissue damping and overall respiratory resistance, either by fluid accumulation or compression of the airways, the involvement of other parameters (e.g., alteration in lung surfactant production) cannot be ruled out.

The role of KvLQT1 channels was also studied under conditions inducing edema in the lung. In WT mice, acute exposure to thiourea (4 h) resulted in a change in the shape of the PV curves, with a statistically significant decrease in static compliance and lung edema flooding, indicated by a >50% increase in water lung content (Figures 3A, B). This observation is in agreement with previous reports (Hesse and Loosli, 1949; Cunningham and Hurley, 1972; Havill and Gee, 1987; Egli et al., 2004) showing the development of lung edema, peaking at ∼4 h. We are aware that this water lung content elevation could be secondary, at least in part, to interstitial fluid accumulation. Indeed, the presence of alveolar fluids was not visible on our histological images of lung sections from thiourea-challenged mice. This could be due to a limitation related to our protocol of hematoxylin and eosin staining of 5 µm sections of embedded tissues, observed with a light microscope with a ×20 objective, which is not well adapted for the observation of alveolar liquid, as confirmed by the CRCHUM pathologist we consulted. Nevertheless, the flooding of lung edema within both the interstitial and alveolar compartments has been previously well described (Hesse and Loosli, 1949; Cunningham and Hurley, 1972; Havill and Gee, 1987; Egli et al., 2004). Using 1 µm sections of Araldite-embedded lung tissues, Cunningham *et al*. (Cunningham and Hurley, 1972) observed the first signs of developing edema in the perivascular space, at 2 h after thiourea injection, followed at 3–4 h by edema fluid infiltration within alveoli. Similarly, histological examination of 1 µm sections of Epon-embedded lungs stained with toluidine blue revealed alveolar edema 4 h after thiourea administration (Egli et al., 2004). Havill et al. (Havill and Gee, 1987) also reported the flooding of edema fluids in the perivascular and alveolar compartments of thiourea animals, peaking at 2–4 h, and which initial rapid removal was preferentially driven through alveolar reabsorption.

Although the KO mice exhibited elevated basal water lung content in physiological conditions, the lung weight analyses following thiourea exposure demonstrated a similar edema index as the WT control mice (exhibiting functional KvLQT1 channels) or the WT ones treated with the KvLQT1 inhibitor. Similarly, lung function parameters were not worsened in KO mice (Supplementary Figure S4). A compensatory effect through other types of K^+^ channels (indicated by the remaining K^+^ conductance in KO mice) may explain this observation.

Importantly, lung treatment with R-L3 significantly reduced the lung edema induced by thiourea in WT mice (Figure 4A left panel). This beneficial effect of R-L3 was prevented by a co-treatment with the chromanol inhibitor or in KO mice lacking the KCNQ1 α-subunit middle and right panels, confirming the specificity of the R-L3 effect through KvLQT1 channels. To the best of our knowledge, this is the first evidence of KvLQT1 channel involvement in lung edema resolution using an *in vivo* model. Nevertheless, Sakuma *et al.* previously reported that an opener of another type of K^+^ channel (i.e. K_ATP_) enhanced the K^+^ transport and alveolar fluid clearance in human resected lungs (Sakuma et al., 1998). These data are in agreement with our previous *in vitro* studies (Leroy et al., 2006; Bardou et al., 2012) showing improved fluid absorption through polarized primary cultures of alveolar epithelial cells after treatment with K_ATP_ and/or KvLQT1 activators.

The measurements of respiratory mechanics done in the present study showed that lung administration of the KvLQT1 activator did not reverse the effect of thiourea on the shape of the PV loop and quasi-static compliance (*C*_*st*_) at the dose and time studied (Figure 4C).

As expected, our data (Figure 4B) confirmed that the thiourea challenge was associated with an increase in lung vascular permeability, due to endothelial barrier damage (Cunningham and Hurley, 1972; Giri et al., 1991). Evans blue extravasation was similar in R-L3/TU mice, indicating that the benefits of KvLQT1 activation on lung edema were not due to a protective effect on the endothelial barrier. Expression and function of various types of KCa, K_ATP_, and Kv channels, including members of the Kv7/KCNQ family, have been observed in endothelial and/or vascular smooth cells (Chatterjee et al., 2008; Ng et al., 2010; Haick and Byron, 2016; Simonsen et al., 2017). However, direct evidence of KvLQT1 function in endothelial integrity, using specific pharmacological drugs, has, to the best of our knowledge, never been reported. Nevertheless, inhibition of vascular Kv7 channels, after intravenous administration of the broad KCNQ blocker linopirdine reduced fluid resuscitation requirements and tissue edema formation after hemorrhagic shock (Nassoiy et al., 2018). On the contrary, Khimenko *et al.* showed that K_ATP_ activation both protected against, and reversed, the increase in endothelial permeability associated with ischemia-reperfusion injury in a model of isolated rat lungs (Khimenko et al., 1995).

Although previous ultrastructural examination under electron microscopy after thiourea exposure (Michel, 1985) showed lung microvascular abnormalities, these are not detectable under light microscopy. Our histological analyses of lung parenchymal sections neither revealed any differences in alveolar structures between our experimental groups (PBS/PBS, PBS/TU, and R-L3/TU conditions, Figure 4D). Immunofluorescence assays however showed a significant reduction in ATI (podoplanin and AQP5) and ATII (pro-SPC) specific cell markers as well as a trend in ENaC reduction (Figures 5A–C, Figure 6A). These results indicate an alteration of alveolar epithelial integrity and/or functionality after the thiourea challenge. It might be postulated that the decrease in water channel AQP5 expression may contribute to lung liquid accumulation in thiourea-treated mice. However, models of acute lung injury (including the one induced by thiourea) in AQP-KO mice, indicated that these water channels are not essential for lung liquid clearance (Song et al., 2000). The decrease in pro-SPC levels, as well as lung edema formation, could both contribute to the altered compliance observed in thiourea-treated mice (Figure 3B).

Despite the R-L3 treatment, pro-SPC levels remained low in thiourea-challenged mice (Figure 5C). A relationship between alveolar surfactant protein expression and the function of another class of K^+^ channel (K2P) has however been shown in a model of lung injury induced by a combination of hyperoxia and mechanical ventilation (Schwingshackl et al., 2017). Importantly, KvLQT1 activation with R-L3 reversed the decrease in AQP5 expression induced by thiourea (Figure 5B). The same pattern was also observed in ENaC levels (although the difference was not statistically significant, Figure 6A). KvLQT1 activation also induced a significant up-regulation of Na^+^/K^+^-ATPase expression in alveolar cells isolated from R-L3/TU-treated mice (Figure 6B). In control experiments, we also defined the level of expression of podoplanin, AQP5, ENaC, and Na^+^/K^+^-ATPase in alveolar cells isolated from mice treated with R-L3, without any thiourea challenge. As shown in Supplementary Figure S5, quantitative ICY analyses of immunostaining assays did not reveal any changes in podoplanin and AQP5 levels, whereas a rising trend in Na^+^/K^+^-ATPase and a significant increase in ENaC were observed. In agreement with our results, one of our previous *in vitro* studies showed that KvLQT1 activation was associated with an increase in α-ENaC promoter activity (through regulation of MAPK activity), a rise in ENaC expression and function, and subsequently, improved liquid absorption through primary alveolar epithelial cell cultures (Leroy et al., 2006; Bardou et al., 2012). Sakuma et al. also reported an increase in alveolar fluid clearance after K_ATP_ channel activation, mediated by amiloride-sensitive sodium channels (Sakuma et al., 1998). Moreover, a rise in Na^+^/K^+^-ATPase expression, after gene transfer, was shown to reduce the wet-to-dry ratio in thiourea-treated mice (Stern et al., 2000). Altogether, the up-regulation of AQP5, α-ENaC, and Na^+^/K^+^-ATPase alveolar expression, secondary to KvLQT1 activation, may contribute to the liquid clearance after thiourea-induced lung edema. It could be thus postulated that the beneficial effect of the KvLQT1 activator on the resolution of lung edema may mainly be driven by the action of alveolar cells, rather than interstitial fluid removal, as indicated by an absence of endothelial preservation by the R-L3 treatment.

**FIGURE 7:**
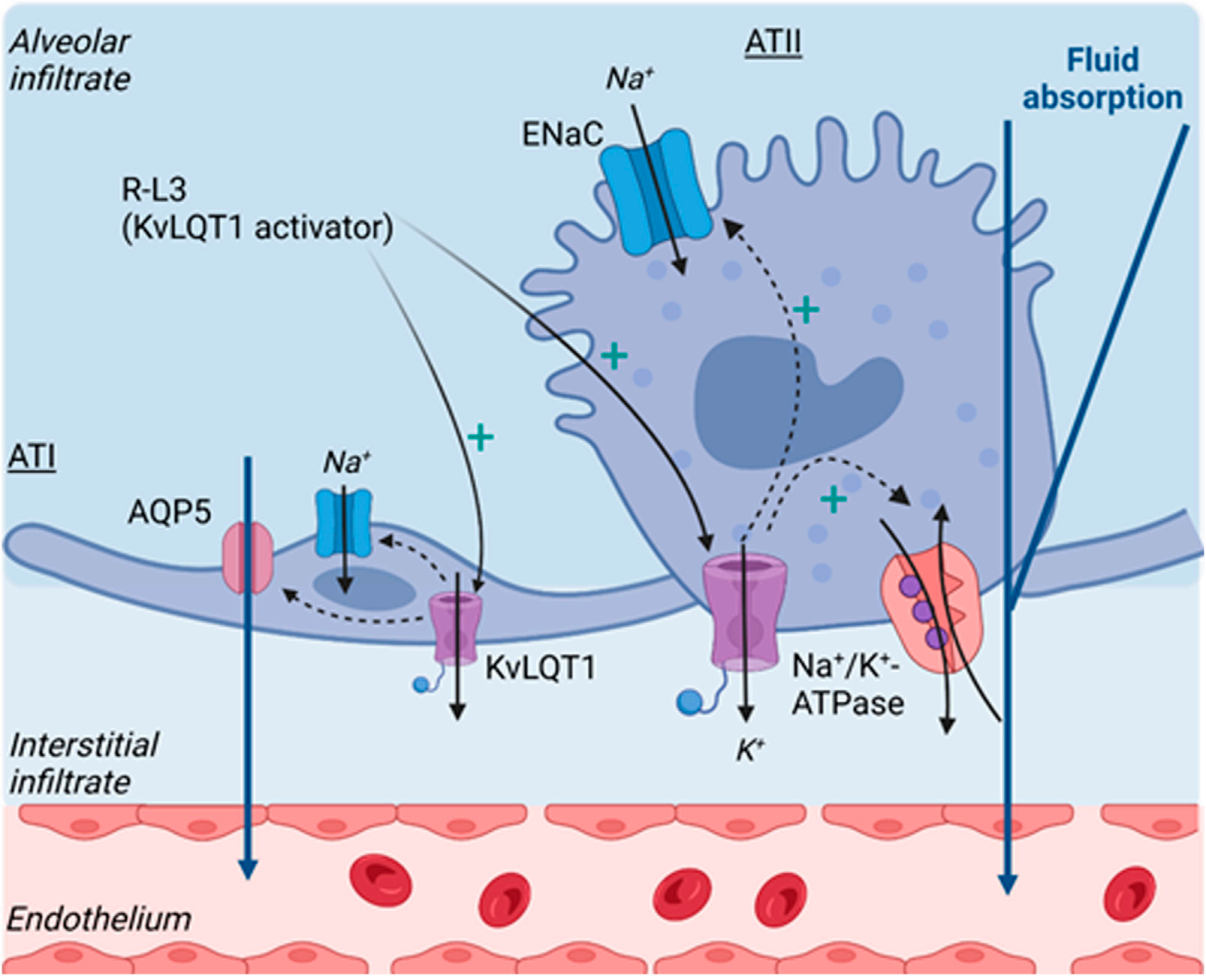
Schematic model describing the beneficial effect of KvLQT1 activation, by R-L3, on lung edema resorption after thiourea challenge, through the observed up-regulation of ion/water channels/transporter involved in sodium and fluid absorption by the alveolar epithelium. Created by Biorender.com.

In summary, our study demonstrated that KvLQT1 extinction slightly altered alveolar liquid homeostasis in physiological conditions but did not worsen lung edema in thiourea-treated mice. The relative contribution, and compensatory effects, of other classes of K^+^ channels (e.g., K_ATP_, K2P, KCa) would need further investigations. Nevertheless, our data indicated a beneficial effect of KvLQT1 activation on lung edema clearance, most probably through the observed up-regulation of channels/transporters involved in alveolar ion/water absorption (as represented in the schematic model in Figure 7).

We are aware that the model of thiourea-induced lung edema recapitulates only part of the main features of acute respiratory distress syndrome (characterized by endothelial vascular and epithelial alveolar damage, exacerbated inflammatory response, and lung edema). Therefore, the role of KvLQT1 channels in the resolution of ARDS parameters remains to be confirmed using complementary models of acute lung injury mimicking direct or extra-pulmonary causes of ARDS. Moreover, other types of K^+^ channels, including stretch-activated channels, have been proposed as therapeutic targets for ARDS (Schwingshackl, 2016). Combined treatments, targeting KvLQT1, K_ATP_, and/or K2P channels, might also be considered.

## Contribution to the field

The importance of ion transport, in particular Na^+^ absorption through ENaC and Na^+^/K^+^-ATPase, for the maintenance of alveolar fluid homeostasis has been well established in physiological conditions as well as after lung edema. There is also some evidence that another class of ion channels, i.e. K^+^ channels participate in the control of ion and liquid transport through the alveolar epithelium. However, the function of K^+^ channels, especially of KvLQT1, has not been studied before *in vivo*. We thus decided to employ complementary approaches using pharmacological drugs, as well as KvLQT1 KO mice, to assess KvLQT1 function in naïve animals and in a model of thiourea-induced lung edema. Our data did not reveal any major lung phenotypic changes in KvLQT1 KO mice in physiological conditions, except for a small accumulation in water lung content. In the thiourea model, KvLQT1 activation favored lung edema resolution and enhanced the expression of ion and liquid channels and transporters (ENaC, Na^+^/K^+^-ATPase, and AQP5) involved in the control of alveolar fluid clearance. These findings highlighted the role of KvLQT1 in lung homeostasis and indicated that these channels may be therapeutic targets after lung edema.

## Data Availability

The raw data supporting the conclusion of this article will be made available by the authors, without undue reservation.
