# Supplementary material for: Impact of KvLQT1 potassium channel modulation on alveolar fluid homeostasis in an animal model of thiourea-induced lung edema
Source: Front Physiol. 2023 Jan 9;13:1069466. doi: 10.3389/fphys.2022.1069466 (PMC9868633; doi:10.3389/fphys.2022.1069466)
Supplement: Supplementary file 1 [file DataSheet1.PDF]

Supplementary Material - Supplementary Figures

Supplementary Figure 1

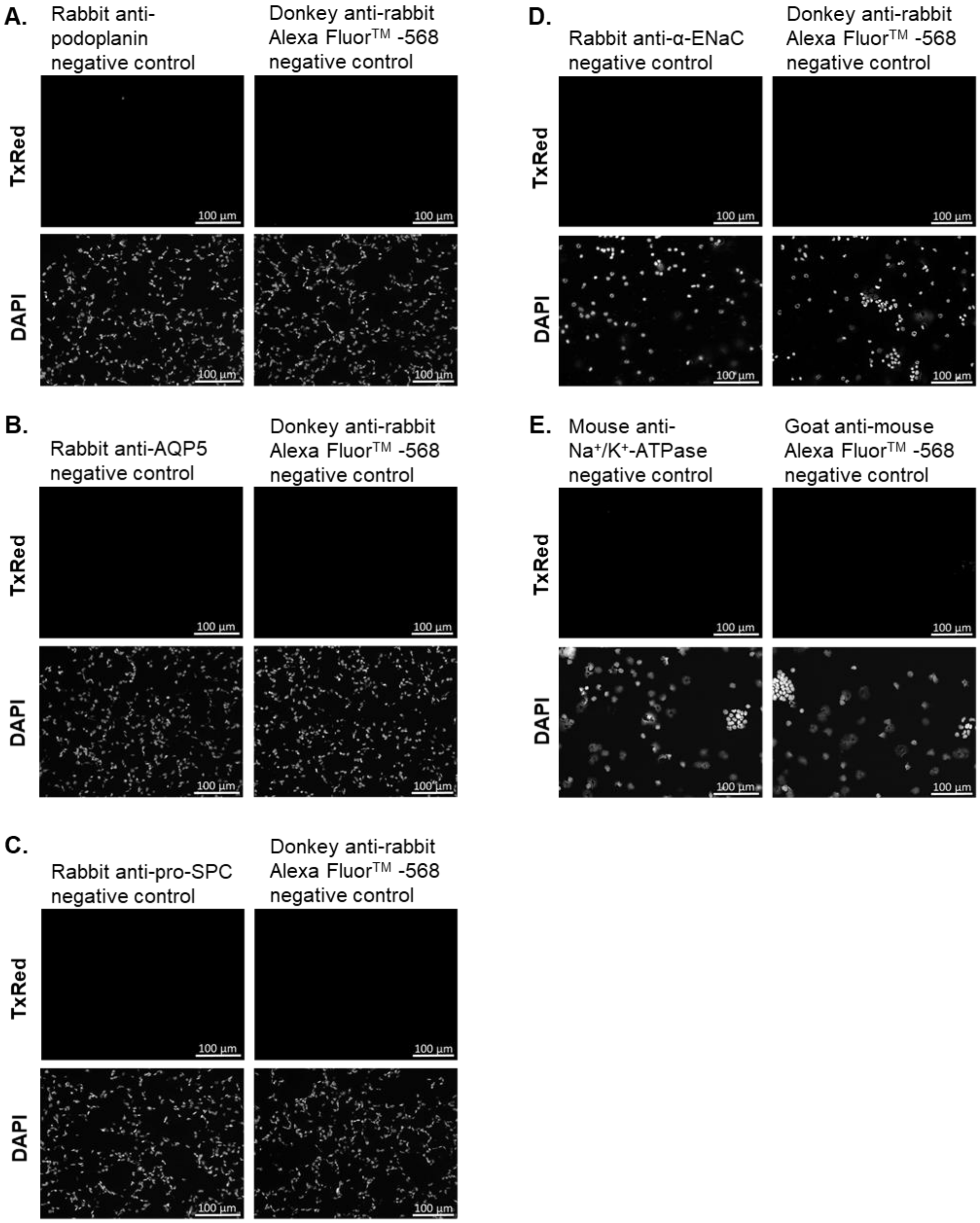

<u>Supplementary Figure 1:</u> **Negative control assays demonstrating the specificity of primary and secondary antibodies used for immunostaining of podoplanin, AQP5, pro-SPC, ENaC, and Na^+/K^+-ATPase.** Representative immunofluorescence images of lung sections (5 µm, Scale: 100 µm) (from WT mice), embedded with paraffin/cryomatrix (**A**, **B**, **C**) and cytocentrifuged ATII cells (**D**, **E**) showing an absence of non-specific signal, after staining with anti-podoplanin (**A**), anti-AQP5 (**B**), anti-pro-SPC (**C**), anti-α-ENaC (**D**) and anti-Na^+/K^+-ATPase (**E**), in absence of secondary antibodies. An absence of background is also confirmed in immunostaining assays of the corresponding secondary antibodies (anti-rabbit Alexa Fluor ™-568 or anti-mouse Alexa Fluor ™-568) alone (in absence of the primary antibody). Nuclei were stained by DAPI.

Supplementary Figure 2

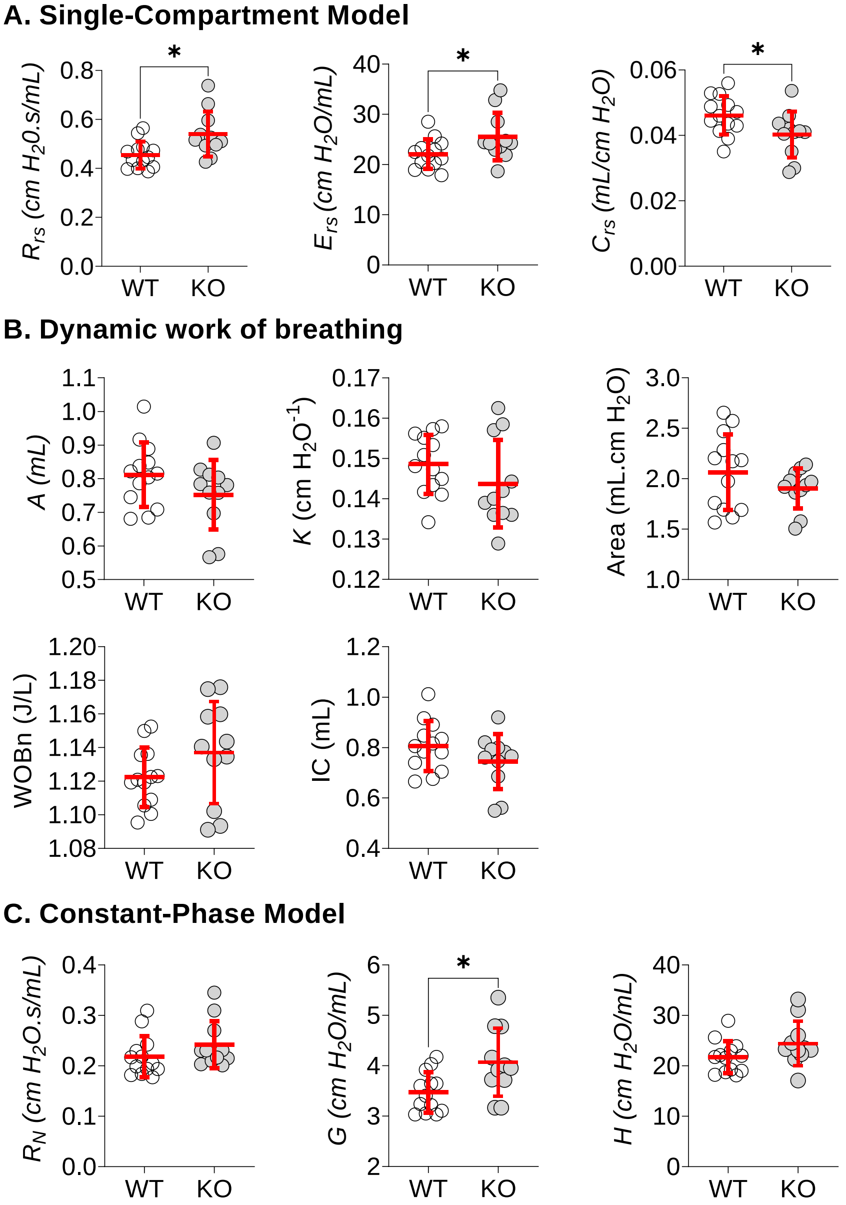

Supplementary Figure 2: Lung function parameters in naïve adult WT and KvLQT1-KO mice. Parameters are presented according to the measurement maneuvers/mathematical model from which they are derived: The single-compartment model (**A**) yields the total respiratory resistance (R_{rs}), elastance (E_{rs}) and compliance (C_{rs}). Partial step-wise PV loops (**B**) give rise to an estimate of the subject's inspiratory capacity (*A*), a shape parameter describing the form of deflating PV-loop (*K*), the area between the PV inflation and deflation limbs (Area), and the inspiratory work-of-breathing normalized to maximal pressure (WOBn). The inspiration capacity (IC) can also be extracted from the Deep Inflation maneuver as the volume at 30 cmH_2O. The constant-phase model (**C**) outputs the Newtonian (airway) resistance (R_N), tissue damping (*G*), and tissue elastance (*H*) parameters. All measurements were made with a flexiVent system in anesthetized and mechanically ventilated naïve adult WT and KvLQT1 KO mice (n=11-13). Results are reported by means ± SEM. Unpaired t-test (Agostino/Pearson normality test: positive) was practiced. *$p < 0.05$ *vs* WT mice.

Supplementary Figure 3

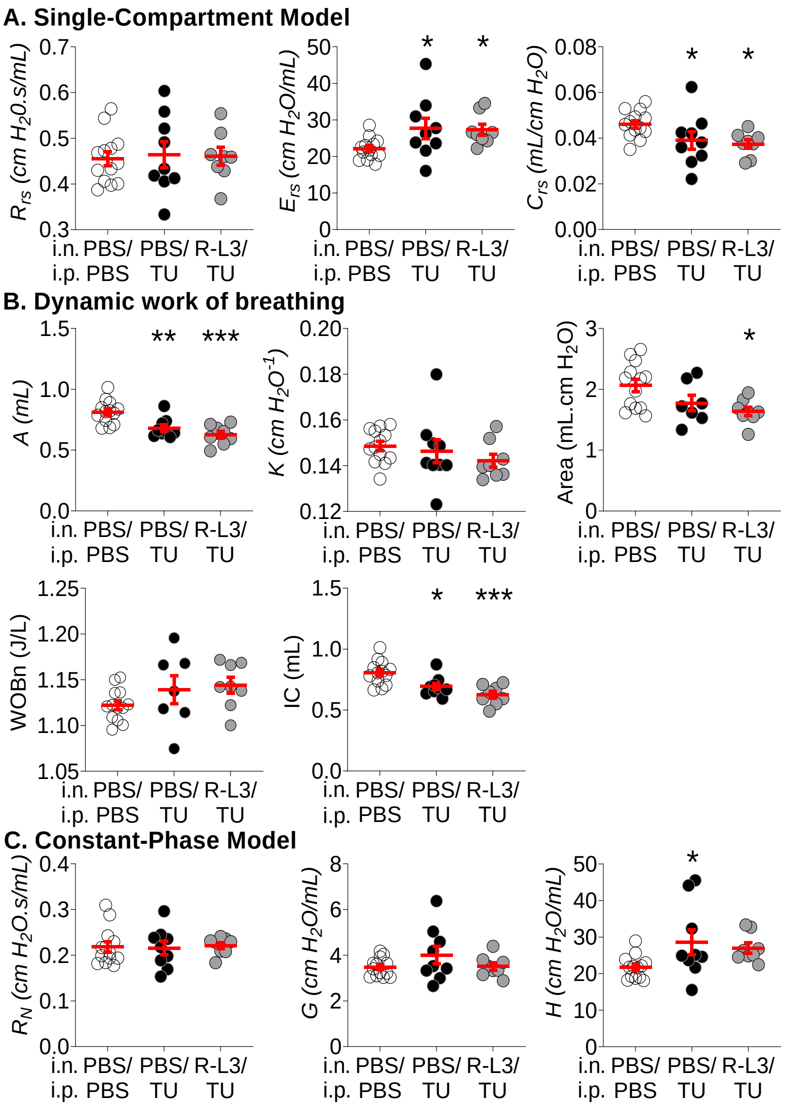

<u>Supplementary Figure 3:</u> Effect of R-L3 on lung function in WT mice after a thiourea challenge. Parameters are presented according to the maneuvers/mathematical model from which they are derived: The single-compartment model (**A**) yields the total respiratory resistance (R_{rs}), elastance (E_{rs}), and compliance (C_{rs}). Partial step-wise PV loops (**B**) give rise to an estimate of the inspiratory capacity (*A*), a shape parameter describing the form of deflating PV-loop (*K*), the area between the PV inflation and deflation limbs (Area), and the inspiratory work-of-breathing normalized to maximal pressure (WOBn). The inspiration capacity (IC) can also be extracted from the Deep Inflation maneuver as the volume at 30 cmH_2O. The constant-phase model (**C**) outputs the Newtonian (airway) resistance (R_N), tissue damping (*G*), and tissue elastance (*H*) parameters. All measurements were made 4 hours after the thiourea challenge with a flexiVent in anesthetized and mechanically ventilated adult WT mice under control conditions (PBS/PBS) or challenged with thiourea (i.p.: TU, 5 mg/kg, PBS/TU) and treated or not with the KvLQT1 activator R-L3 (R-L3/TU) (n=5-9). Results are reported by means ± SEM. One-way ANOVA and Bonferroni's multiple comparisons test were practiced (normality Agostino/Pearson test: positive) was practiced. *$p < 0.05$ or *** $p < 0.0001$ *vs* PBS/PBS.

Supplementary Figure 4

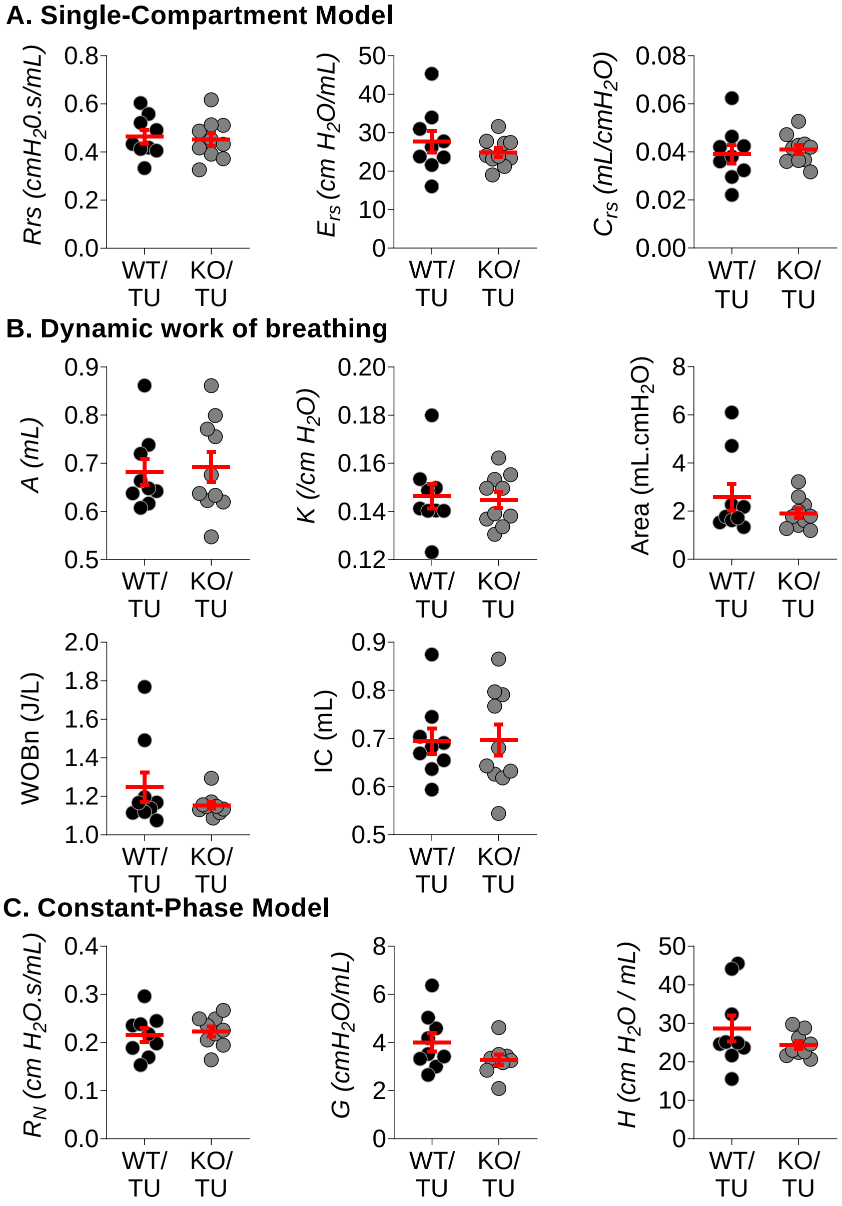

<u>Supplementary Figure 4:</u> Lung function parameters in WT and KO mice after a thiourea challenge. Parameters are presented according to the maneuvers/mathematical model from which they are derived: The single-compartment model (**A**) yields the total respiratory resistance (R_{rs}), elastance (E_{rs}) and compliance (C_{rs}). Partial step-wise PV loops (**B**) give rise to an estimate of the inspiratory capacity (*A*), a shape parameter describing the form of deflating PV-loop (*K*), the area between the PV inflation and deflation limbs (Area), and the inspiratory work-of-breathing normalized to maximal pressure (WOBn). The inspiration capacity (IC) can also be extracted from the Deep Inflation maneuver as the volume at 30 cmH_2O. The constant-phase model (**C**) outputs the Newtonian (airway) resistance (R_N), tissue damping (*G*) and tissue elastance (*H*) parameters. All measurements were measured made 4 hours after the thiourea challenge with a flexiVent in WT and KO mice (i.p.: TU, 5 mg/kg, WT/TU vs KO/TU, n=9-10). Results are reported by means ± SEM. All parameters were comparable between WT and KO animals.

Supplementary Figure 5

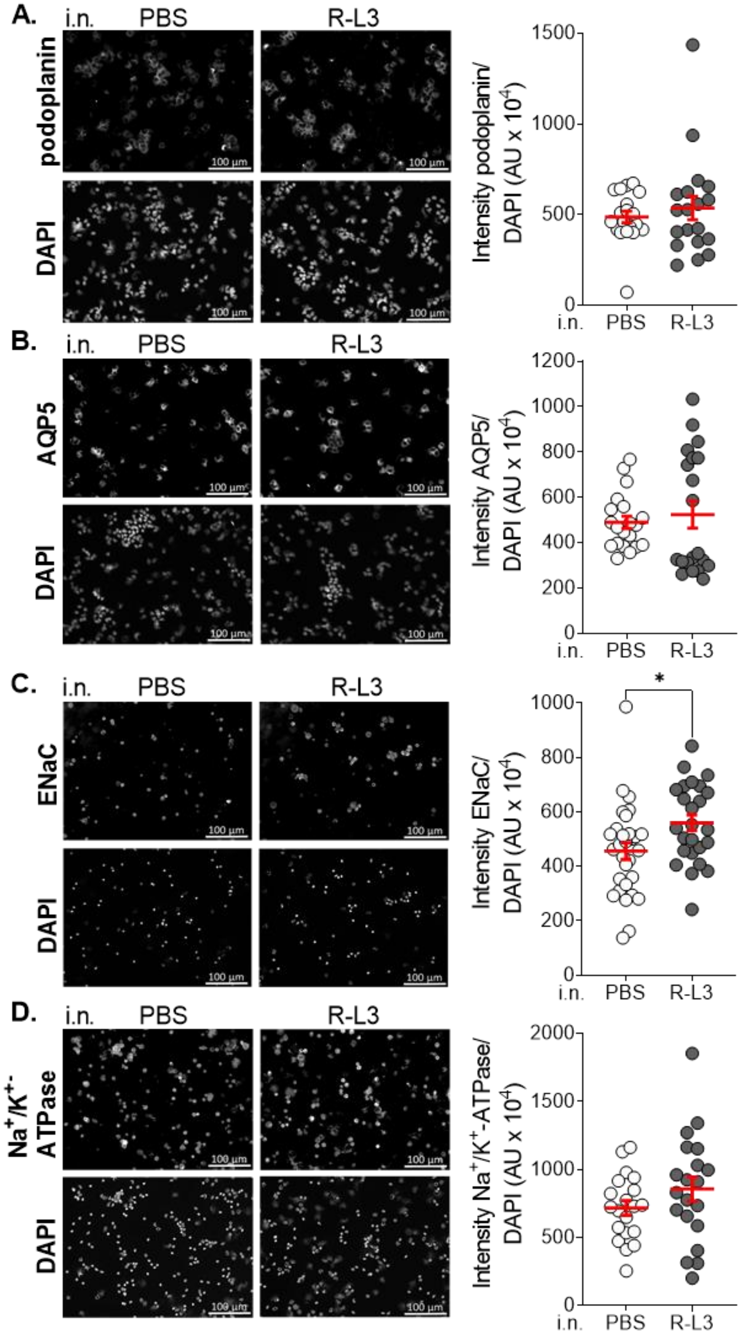

<u>Supplementary Figure 5:</u> Effect of R-L3 treatment on the expression of alveolar markers and ion/liquid channels/transporters in absence of thiourea challenge. Representative immunofluorescence images (Scale: 100 µm) of podoplanin (**A**), AQP5 (**B**), α-ENaC subunit (**C**), and Na^+/K^+-ATPase (**D**) stainings of slides with cytocentrifuged ATII cells isolated from WT control mice (PBS), treated with the KvLQT1 activator (R-L3, 4µM) for 24 hours before lung collection (n=2 experiments, each including a pool of 4-9 mice). Nuclei were stained by DAPI. Quantification (right panels) of all staining intensities was made with a protocol exploited by ICY Software. Values are presented as means ± SEM. Unpaired t-test (Agostino/Pearson normality test: positive). $^*p < 0.05$ *vs* PBS.
